# Open pelvic fracture: the killing fracture?

**DOI:** 10.1186/s13018-018-0793-2

**Published:** 2018-04-13

**Authors:** E. Hermans, M. J. R. Edwards, J. C. Goslings, J. Biert

**Affiliations:** 1 Department of Surgery, Radboud University Medical Center, Nijmegen, PO Box 9101, 6500 HB Nijmegen, The Netherlands; 2 Department of Surgery, Onze Lieve Vrouwe Gasthuis Amsterdam, PO-BOX 95500, 1090 HM Amsterdam, The Netherlands

## Abstract

**Background:**

Open pelvic fractures are rare but represent a serious clinical problem with high mortality rates. The purpose of this study was to evaluate the outcomes of open pelvic fractures in our clinic and to compare the results from our patient group with those of closed fractures and with the literature from the past decade.

**Methods:**

Data of patients older than 16 years of age who were admitted to our hospital with a pelvic fracture between January 1, 2004, and December 31, 2014, were analyzed. The collected data were patient demographics, mechanism of injury, RTS, ISS, transfusion requirement during the first 24 h, Gustilo-Anderson and Faringer classification, number and type of interventions complications, mortality, and length of stay.

**Results:**

Twenty-four of 492 patients (5% of all pelvic fracture patients) had an open fracture. Their mean age was 36 years, the mean ISS was 31, and the mean number of transfused packed red blood cells was 5.5. These numbers were all significantly higher than in the patients with a closed fracture, although they were comparable to other studies with open fractures.

The mortality was 4% in the open group versus 14% in the closed group (*p* = 0.23). The reported mortality in the literature ranges between 4 and 45%.

**Conclusion:**

Open pelvic fractures are relatively rare but are a cause of significant morbidity. In this series, we treated patients with open pelvic fractures successfully, with a survival rate of 96%. There was no significant difference in survival rate between open and closed pelvic fractures. Compared with other studies, the mortality in our study was relatively low.

## Background

**Fig. 1:**
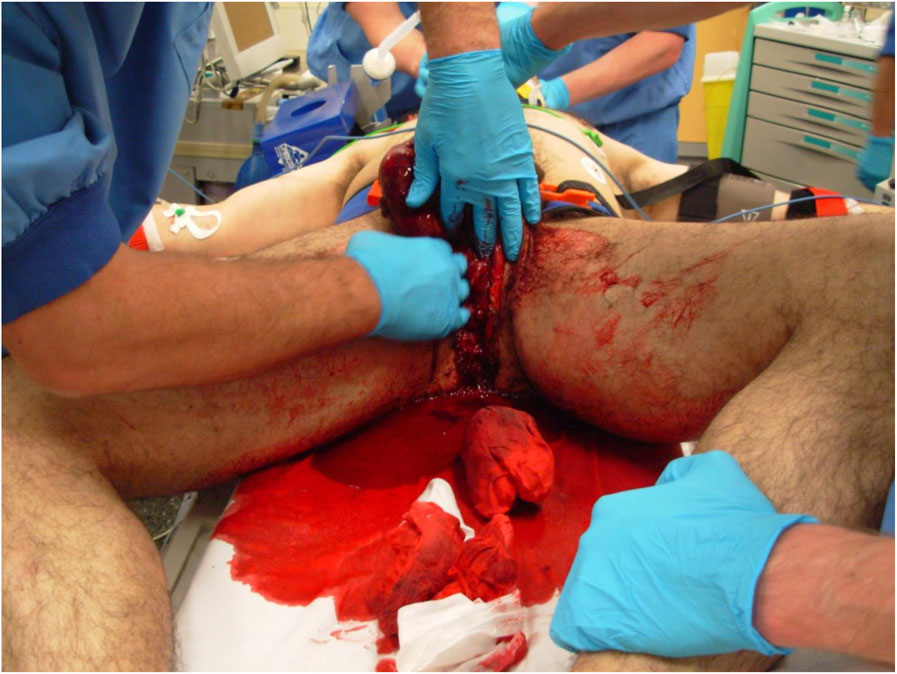
Open pelvic fracture with massive hemorrhage. Initial packing in the emergency department. A pelvic binder is already applied

Pelvic fractures are often caused by high-energy trauma, and these patients often have multiple injuries. Open pelvic fractures are rare, with an estimated incidence of 2–4% of all pelvic fractures [1]. Open pelvic fractures are characterized by direct communication between the fracture hematoma and the external environment (through the rectum, vagina, or skin). Patients are at risk for early exsanguination because massive hemorrhage can occur due to disruption of the natural anatomic compartment and loss of the tamponade effect (Fig. 1). Late mortality is caused by pelvic sepsis and multiple organ failure [2]. Historically, mortality rates up to 50% were reported in the 1970s [3], which was considerably higher than the mortality rates reported for closed fractures in the same period [4]. In the 1990s, there was an improvement in treating these injuries. Leenen et al. [5] reported a mean ISS of 48 and mortality rate of 14.3% in their open pelvic fracture group. Other studies from the 1990s also reported mortality rates of 15–30% [1]. However, some authors have even reported mortality rates as low as 5% [6, 7]. This decline was set in motion by new aggressive trauma protocols including damage control surgery, fecal diversion, a multidisciplinary team approach, and advances in critical care.

The purpose of this study was to evaluate the outcomes of open pelvic fractures in our clinic and to compare the results from our patient group with those of closed fractures and with the literature from the past decade.

## Methods

The Radboudumc Nijmegen (RUMCN) is a level 1 trauma center and an expert center for pelvic and acetabular fractures in the Netherlands.

All data were analyzed from our electronic database. All patients who arrived alive at the RUMCN with a pelvic fracture between January 1, 2004, and December 31, 2014, were included if they were 16 years or older and were admitted to our clinic. The following data were collected: patient demographics, mechanism of injury, vital signs in the emergency room (ER), Glasgow Coma scale (GCS) score in the ER, Revised Trauma Score (RTS), Abbreviated Injury Scale (AIS), Injury Severity Score (ISS), fracture classification according to Tile [8], severity of soft tissue damage according to Gustilo and Anderson [9], injury zone classification according to Faringer [10], concomitant injuries, amount of blood products administered during the first 24 h and/or intravenous fluids (colloid, crystalloid), treatment in the ER, operative treatment, number of surgical interventions, infectious complications within 30 days after admission as recorded in the patient chart, length of stay (LOS), mortality, cause of death, time of death after the accident, and destination after discharge. Urogenital complaints during follow-up as well as consolidation of the fracture on X-ray or CT were noted.

**Fig. 2:**
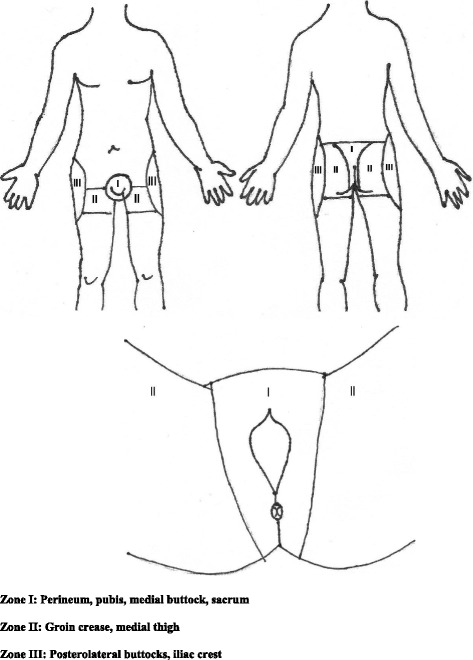
The Faringer classification

An open pelvic fracture was defined as a fracture with a direct connection between fracture surfaces and the external environment (through the skin, rectum, or vagina). Patients were divided in two groups: one group with open pelvic fractures (OG), and one group with closed pelvic fractures (CG). Figure 2 illustrates the Faringer classification. We considered bowel injuries and vaginal wounds to be zone 1 lesions.

A pelvic sepsis was defined as a sepsis caused by intrapelvic abscesses which required percutaneous drainage.

### Literature search

To compare our results with recent literature, a literature search of all studies regarding the outcome of open pelvic fractures from 2005 to 2017 was done in PubMed. The following MeSH terms were used: (open[All Fields] AND (“pelvis”[MeSH Terms] OR “pelvis”[All Fields] OR “pelvic”[All Fields]) AND (“fractures, bone”[MeSH Terms] OR (“fractures”[All Fields] AND “bone”[All Fields]) OR “bone fractures”[All Fields] OR “fracture”[All Fields]) AND outcome[All Fields]). In total, 233 articles were found. After reading the abstracts, only seven articles were eligible for our study.

### Treatment protocol

Patients were treated in the trauma room according to ATLS® guidelines [11]. When patients were in severe hemorrhagic shock, principles of damage control resuscitation were applied, and our massive transfusion protocol was initiated. Since 2014, the early administration of tranexamic acid, as advocated in the CRASH-2 trial by Roberts et al. [12], has been added to our resuscitation protocol. A pelvic binder was applied immediately when a pelvic fracture was suspected. A CT scan was performed in all patients, except for patients who were non-responders to initial therapy. These patients were directly moved to the operating theater. A full physical examination was performed on each patient, including a perineal and vaginal exam if a pelvic fracture was suspected. All patients with open fractures received tetanus prophylaxis and antibiotics. Patients with a grade I open fractures, according to Gustilo and Anderson, received cephazolin IV once. All grade II or higher open fractures were treated with cephazolin IV for at least 5 days and aggressive surgical debridement and temporary closure with vacuum assisted closure (VAC) therapy or, if feasible, primary closure of the traumatic pelvic wound. When patients had a rectal injury or a massive perineal injury, an emergency laparotomy was performed and a colostomy was placed for fecal diversion. When patients had a urethral or bladder injury, the urologist was consulted, and a suprapubic catheter was inserted. Operative stabilization of the fracture was performed in unstable pelvic fractures (Tile B1 and 3, Tile C1-3) according to damage control principles. Throughout our study period, this protocol remained the same.

### Statistical analysis

All statistical analyses were performed with SPSS© statistical software version 22.0 (SPSS, Chicago, IL). We calculated the *p* values using the independent samples *t* test or Mann-Whitney test to compare means and the chi-square test for categorical variables. A *p* value of < 0.05 was considered significant.

## Results

Between January 2004 and December 2014, 537 patients with a pelvic ring fracture were admitted to the RUMCN. We excluded 48 children. The data of 492 patients were analyzed. Twenty-four of 492 patients had an open pelvic fracture (5%).

### Demographics and trauma severity

Patient characteristics and trauma severity scores are listed in Table 1. The male:female ratio in both groups was approximately 3:1. All patients in the OG suffered high-energy trauma (HET) compared with 87% in the CG (*p* < 0.01). The mechanisms of injury are listed in Table 2.

**Table 1:** Patient characteristics

	Open Average ± SEM	*n* = 24 Range	Closed Average ± SEM	*n* = 468 Range	*p*
Age	36 ± 2.7	17–58	43 ± 1.2	16–90	0.008
Male (%)	17 (71)		311 (66)		0.88
RTS	11 ± 0.3	7–12	10 ± 0.15	4–12	0.38
ISS	31 ± 4.4	9–66	26 ± 1	9–75	0.008
Shock class > 3	11		119		0.03
PRBCs (< 24 h)	5.5 ± 4.2	0–30	3.5 ± 0.4	0–34	0.004

*PRBCs* packed red blood cells

**Table 2:** Mechanism of injury

	Open (*n* = 24) No. of patients (%)	Closed (*n* = 468) No. of patients (%)	*p*
High-energy trauma	24 (100)	407 (87)	0.01
Low-energy trauma	0 (0)	61 (13)	
Trauma mechanism			
Traffic accident	19 (80)	265 (65)	
Fall from height	2 (8)	106 (26)	
Crush injury	3 (12)	37 (9)	

Patients with an open fracture were significantly younger (mean 36 vs 43 years *p* = 0.008), had a higher ISS (mean 31 vs 26 *p* = 0.008), were more likely to present a shock class of 3 or higher (*p* = 0.03), and received significantly more packed red blood cell units (PRBCs) during the first 24 h (mean 13.2 vs 4.1 *p* = 0.004). The Tile classifications for both groups are listed in Table 3. Tile C fractures were more frequently observed in the OG (*p* < 0.01). Table 4 lists all patients with open fractures.

**Table 3:** Tile classification

		Open (*n* = 24) No. of patients (%)	Closed (*n* = 468) No. of patients (%)
A		7 (29)	108 (23)
	A1	0	0
	A2	6	92
	A3	1	16
B		5 (21)	140 (30)
	B1	2	32
	B2	1	87
	B3	2	21
C		12 (50)	220 (47)
	C1	6	139
	C2	3	35
	C3	3	46

Associated injuries were frequently observed in both groups, as reflected by a high ISS in both groups. Only 3 patients (12.5%) in the OG had no other injuries than the open pelvic fracture. In the CG, 45 patients (9.5%) had no other injuries. In both groups, additional injuries to the chest and extremities were most often encountered. Additional injuries are listed in Table 5.

**Table 4:** Patients with open fractures

No.	Sex	Age	MOI	ISS	Tile	Location	Far	GA	LOS	Col	Survivor
1	M	42	Crush	4	A	Iliac wing	3	I	5	N	Y
2	M	24	MVA	41	C	Buttocks	2	II	47	N	Y
3	M	45	MVA	29	B	Rectum	1	II	30	Y	Y
4	F	27	MVA	45	C	Vulva	1	II	26	N	Y
5	M	47	MVA	38	A	Small bowel	3	IIIa	96	N	Y
6	M	44	MVA	38	C	Perineum	1	II	55	Y	Y
7	M	39	MVA	34	B	Rectum	1	IIIa	12	Y	Y
8	M	38	MVA	66	C	Iliac wing	3	IIIa	59	N	Y
9	F	19	MVA	16	C	Anal cleft	1	II	16	Y	Y
10	M	58	MVA	38	B	Perineum	1	II	25	Y	Y
11	M	27	MVA	59	B	Perineum	1	IIIa	71	Y	Y
12	M	27	MVA	22	A	Iliac wing	3	I	47	N	Y
13	F	20	Crush	48	C	Perineum	1	I	29	N	Y
14	M	42	MVA	9	A	Iliac wing	3	I	0	N	Y
15	M	44	MVA	38	C	Groin	2	II	12	N	Y
16	M	55	FFH	33	C	Iliac wing	3	I	44	N	Y
17	M	39	MVA	13	A	Buttock	2	II	9	N	Y
18	F	36	Crush	4	C	Groin	2	I	7	N	Y
19	F	17	MVA	57	C	Vagina	1	I	59	N	Y
20	F	42	FFH	32	C	Rectum	1	II	42	Y	N
21	M	47	MVA	29	A	Abdominal wall	3	IIIA	67	N	Y
22	M	19	MVA	14	A	Abdominal wall	3	II	4	N	Y
23	F	54	MVA	22	B	Iliac wing	3	II	23	N	Y
24	M	19	MVA	16	C	Scrotum	1	II	11	N	Y

*MOI* mechanism of injury, *GA* Gustilo-Anderson classification, *LOS* length of stay (days), *Col* colostomy, *MVA* motor vehicle accident, *Far* Faringer zone, *FFH* fall from height

**Table 5:** Associated injuries

	Open (*n* = 24)	Closed (*n* = 468)
Head and neck	6	85
Chest	16	157
Abdomen	9	85
Spine	6	62
Extremities	17	141

### Treatment of open pelvic fractures

Nine patients were treated with a pelvic binder on scene by the paramedics. An additional 4 patients had a pelvic binder applied in the trauma room on clinical suspicion. In 11 patients with a shock class of 3 or higher and in 4 patients with a shock class of 2, the massive transfusion protocol was initiated.

In 14 patients with an open pelvic fracture, immediate operative stabilization of the pelvic fracture was performed. In 10 patients, open reduction and internal fixation (ORIF) was performed, and in 2 additional patients, this was combined with a pelvic C-clamp. One patient was treated with a pelvic C-clamp only, and in another patient, both a pelvic C-clamp and an external fixator were placed. Ten patients were not treated operatively for their pelvic fractures in the acute phase.

Two additional patients were treated operatively after a period of stabilization in the ICU. In addition, in all patients with an external fixator or C-clamp, internal fixation was performed, either with ORIF or with percutaneous screws.

In 8 patients, the pelvic fractures were treated non-operatively. These were all patients with intrinsic stable pelvic fractures (Tile A and B2). Additional angiography and selective embolization because of persistent hemodynamic instability after operative treatment was successfully performed in 3 patients (12%). In the closed group, selective embolization was done in 20 patients (4%). This was not significantly different between both groups, although a trend towards significance was found in favor of the OG (*p* = 0.09).

### Fecal diversion and pelvic infections

Eleven of our patients had a Faringer zone 1 injury, 4 patients had a zone 2 injury, and 9 patients had a zone 3 injury. The Gustilo-Anderson classification in relation to the Faringer zone is listed in Table 6.

**Table 6:** Relationship between Faringer and Gustilo-Anderson classification

	Faringer 1	Faringer 2	Faringer 3
GA I	2	1	4
GA II	7	3	2
GA III	2	0	3

Of the 11 patients with an injury in Faringer zone 1, 3 patients had lacerations of the scrotum or vagina. They all were treated with debridement and primary closure. No fecal diversion was necessary in these patients.

Eight patients had a rectal or perineal injury; 7 of them underwent fecal diversion. The patient without fecal diversion had a GA I open fracture of the perineum and was treated with wound debridement and antibiotics.

One patient with a Faringer zone 1 injury developed pelvic sepsis. This patient had a type C fracture with perforation of the rectum. The ISS of this patient was 32, and there were additional injuries to the chest and small bowel. A colostomy was placed, and secondary plate fixation of the sacrum was performed on day 6. During admittance, the patient developed a pulmonary embolism and had multiple intra-abdominal and intrapelvic abscesses that required drainage. Despite multiple operative procedures, the patient developed uncontrollable sepsis and died on day 42. None of the other patients with a zone 1 injury developed pelvic sepsis or infectious complications related to the perineal injury.

In patients with zone 2 or 3 injuries, no fecal diversion was performed, in accordance with our protocol. Infectious complications were observed in 4 patients. One patient had multiple small bowel perforations due to osseous fragments perforating the small bowel and developed multiple intra-abdominal abscesses, which required multiple laparotomies for drainage.

Two patients with an open iliac wing fracture developed an infected hematoma and were treated with debridement and VAC-therapy; 1 patient with a groin laceration developed a superficial wound infection, which was treated by opening the closed wound and secondary healing.

### Mortality

Outcomes are listed in Table 7. The total length of stay as well as the length of stay in the ICU was significantly higher in the OG.

**Table 7:** Outcomes

	Open (*n* = 24) No. of days ± SEM	Range	Closed No. of days ± SEM	Range	*p*
LOS	44.1 ± 9.3	4–166	20.3 ± 1.4	0–142	0.021
ICU LOS	15.4 ± 6.1	0–107	6 ± 0.7	0–64	0.032
Mortality no. (%)	1 (4%)		68 (14%)		NS

*LOS* length of stay, *ICU* intensive care unit, *NS* not specified

One patient in the OG died due to uncontrollable sepsis (4%). In the CG, 68 patients died (14%).

### Discharged patients

For the destination after discharge, see Table 8. In the OG, the patients who were discharged home had a significantly lower mean ISS and shock class (*p* = 0.02) and received substantially fewer packed red blood cells during the first 24 h (*p* = 0.1). There were no significant differences in the Tile classification. In the CG, the mean ISS, shock class and number of RBCs admitted during the first 24 h were significantly lower (*p* = < 0.005). The group that was released to home had fewer Tile C fractures (36 vs 58) (*p* = 0.07).

**Table 8:** Destination after discharge

Destination	OG (*n* = 24) No. (%)	CG (*n* = 468) No. (%)
Home	11 (46)	197 (42)
Other hospital	0	94 (20)
Rehabilitation facility	11 (46)	89 (19)
Nursing home	1 (4)	19 (4)
Unknown	0	1 (1)
Deceased	1 (4)	68 (14)

*NS* not significant

### Follow-up

The mean follow-up of the OG was 6 months (range, 6 weeks–4 years). Restoration of continuity of the bowel was performed in 5 patients (71%). Median time to surgery was 4 months (range 6 weeks–1.2 years). The 2 other patients were not deemed fit for surgery. No problems of fecal or urinary incontinence were detected in the OG. In 2 patients, sexual problems were noted; 1 patient had dyspareunia, and 1 patient complained of impotence. Non-unions were not observed during the follow-up.

### Comparison with the literature

Table 9 shows the outcome of studies conducted from 2005 to the present. In total, 7 other studies were identified [13–19]. All studies but one had an inclusion period of 10 years. Most studies encountered 1 to 5 patients per year with an open pelvic fracture. The mean age was 36 years (range 28–41), the mean ISS was 27 (range 21–31.5), and the mean number of transfused PRBCs was 10.5 (range 5.5–17.2). The mortality differed greatly between groups, with a range of 4–45%. The mean mortality rate was 27%.

**Table 9:** Reported outcomes in the literature

Authors	Year of publication	No. of patients	Study period	Mean age	Mean ISS	Mean no of PRBCs	Mortality (%)
This study	2017	24	2004–2014	36	31	5.5	4
Giordano et al.	2016	30	2000–2010	28.4	21	NS	40
Fitzgerald et al.	2014	181	2002–2012	33.6	22.7	7.2	17
Hasankhani et al.	2013	15	2006–2010	38.6	29	8	13.3
Wei et al.	2012	16	2000–2010	41	29	NS	31
Dong et al.	2011	41	2001–2010	32.8	31.4	17.2	24
Black et al.	2011	52	1999–2009	39	23	14	19
Dente et al.	2005	44	1995–2004	39.2	29.6	11.5	45

*PRBCs* packed red blood cells*, NS* not specified

## Discussion

Patients with open pelvic fractures are rare. Most authors that reported on this fracture type encounter this type of injury 2–5 times a year. In a 10-year period, we treated 24 patients with this injury at our level 1 trauma center, which is the largest series reported in the Netherlands.

All patients with open fractures had suffered high-energy trauma, which illustrates the high kinetic forces that are required to develop an open fracture. Because of the high kinetic forces involved, concomitant injuries are high, as was reflected in this study by the high ISS scores (mean 31 in the OG vs 26 in the CG). Compared with other reports, this was relatively high, with only one study reporting a higher mean ISS (31.4) [17].

After the source of hemorrhage is controlled and the patient is adequately stabilized, aggressive wound debridement and irrigation is indicated. According to Woods et al. [20], fecal diversion is only useful in patients with extensive soft tissue injury or posterior wounds.

Faringer et al. [10] advocated that all Faringer zone I open pelvic fractures should undergo fecal diversion. In our study, 11 patients had an injury to Faringer zone 1. Seven patients underwent a diverting colostomy. Infectious complications were only observed in 1 patient, as noted earlier. The patients in whom no colostomy was performed had vaginal or scrotal lesions. These patients were classified as having a Faringer zone 1 injury, but these wounds were no reason for a colostomy. We believe a low threshold should be employed for the use of a diverting colostomy and rectal wash-out in patients with large perineal wounds and rectal injuries. This recommendation is supported by other authors [21, 22].

Although patients in the OG were more severely injured, the mortality from open pelvic fractures did not differ significantly from that in patients with closed pelvic fractures. The trend of higher survival rates in the open group is possibly caused by the relatively small number of open pelvic fractures compared with a large group of closed fractures. Fracture types were comparable to other studies [5, 14, 19]. When comparing our study to seven other studies published in the 1990s [1], the ISS in our patient group was the second highest compared with eight studies, and the mean age was the third highest; however, only one study had a comparable mortality rate [7]. We compared our results to those of more recent studies. The mortality varied greatly. Dente et al. [19] reported a mortality rate of 45%. They reported a higher mean age (39.2), a lower mean ISS (29.6), a lower mean RTS (9.5), and a lower transfusion requirement during the first 24 h (11.5); their male:female ratio was comparable. Forty-three percent had a grade III Gustilo-Anderson injury vs 64% in our study. In the study conducted by Giordano et al. [13], the mean age and ISS were lower, although the mortality was as high as 40%. Their explanation for this high number was the high ISS and a high relationship to Jones type 3 injuries, which are unstable pelvic ring injuries with a concomitant rectal injury [2]. However, the patients in our study had a higher ISS and a comparable number of rectal injuries. Other studies show a comparable mean age, ISS, and transfusion rate. However, some authors only included patients with extensive perineal injuries [15, 16] which may be the cause of the different mortality rates.

It remains unclear why the mortality rates in different studies show so much variation. We feel that the contribution to mortality of the open pelvic fracture by itself can be divided in two distinct effects: massive blood loss because of loss of containment and infectious complications. In both subgroups, an aggressive treatment protocol was shown to be effective in our group of patients, resulting in a low mortality rate. The third contribution to mortality should be found in the high-energy transfer to the rest of the body. Of course, head trauma, chest injuries, and blunt abdominal trauma form a variety of causes for patient demise. In this patient group, time is of the essence. Obtainability of dedicated urgent care in regional trauma centers in the Dutch situation with short prehospital transfer times can be beneficial. This possibly explains why the mortality rates in other studies may be higher.

It can be questioned whether mortality or patient-related outcome measurements (PROMS) should be the endpoint of interest in this type of injury. PROMS are currently being evaluated in a prospective study in our center.

## Conclusion

Although open pelvic fractures are relatively rare, the morbidity and use of resources are higher than in patients with closed pelvic fractures. In our series, however, the mortality was not significantly higher in comparison with closed pelvic fractures. We recommend aggressive surgical debridement and stabilization in patients with open pelvic fractures to prevent ongoing hemorrhage and pelvic sepsis to change this injury from a “killing fracture” to a survivable injury.
